# Photocoagulation or sham laser in addition to conventional anti-VEGF therapy in macular edema associated with TelCaps due to diabetic macular edema or retinal vein occlusion (TalaDME): a study protocol for a multicentric, French, two-group, non-commercial, active-control, observer-masked, non-inferiority, randomized controlled clinical trial

**DOI:** 10.1186/s13063-024-07994-1

**Published:** 2024-04-22

**Authors:** Bénédicte Dupas, Daniela Castro-Farias, Jean-François Girmens, Ali Eginay, Aude Couturier, Frederic Villeroy, Marie-Noëlle Delyfer, Catherine Creuzot-Garcher, Audrey Giocanti-Auregan, Laurence Béral, Carl Arndt, Charles Mesnard, Eric Vicaut, Philippe Chaumet-Riffaud, Isabelle Durand-Zaleski, Michel Paques

**Affiliations:** 1 Service d’ophtalmologie, Hôpital Lariboisière, AP-HP Nord, Université Paris Cité, Paris, France; 2 FRCRnet/FCRIN Network, Paris, France; 3 Hopital Des Quinze-Vingts, Centre d’Investigation Clinique 1423, INSERM, Paris, France; 4 Service d’ophtalmologie, CHU de La Réunion, Hôpital Félix Guyon, Saint-Denis, France; 5 Service d’ophtalmologie, CHU de Bordeaux – Hôpital Pellegrin, Bordeaux, France; 6 FRCRnet/FCRIN Network, Bordeaux, France; 7 Service d’Ophtalmologie, CHU de Dijon – Hopital François Miterrand, Dijon, France; 8 FRCRnet/FCRIN Network, Dijon, France; 9 Service d’Ophtalmologie, Hôpital Avicenne, Université Sorbonne Paris Nord, Bobigny, France; 10 Service d’Ophtalmologie, CHU de Pointe-À-Pitre, Les Abymes, France; 11 Service d’Ophtalmologie, CHU de Reims, Reims, France; 12 Service d’Ophtalmologie, CHU de Martinique, Fort-de-France, France; 13 URC Lariboisière-St Louis, Hôpital Fernand Widal, AP-HP Nord, Université Paris Cité, Paris, France; 14 DRCI URCEco Et Hôpital Henri Mondor, AP-HP, Université Paris Est Créteil, Paris, France

**Keywords:** Macular edema, Diabetic retinopathy, Retinal vein occlusion, Laser, Photocoagulation, Telangiectatic capillaries (TelCaps)

## Abstract

**Background:**

Macular edema (ME) results from hyperpermeability of retinal vessels, leading to chronic extravasation of plasma components into the retina and hence potentially severe visual acuity loss. Current standard of care consists in using intravitreal injections (IVI), which results in a significant medical and economic burden. During diabetic retinopathy (DR) or retinal vein occlusion (RVO), it has recently been shown that focal vascular anomalies (capillary macro-aneurysms, also termed TelCaps) for telangiectatic capillaries may play a central role in the onset, early recurrence, and/or persistence of ME. Since targeted photocoagulation of TelCaps may improve vision, identification, and photocoagulation of TelCaps, it may represent a way to improve management of ME.

**Objective:**

The Targeted Laser in (Diabetic) Macular Edema (TalaDME) study aims to evaluate whether ICG-guided targeted laser (IGTL), in association with standard of care by IVI, allows reducing the number of injections during the first year of treatment compared with IVI only, while remaining non-inferior for visual acuity.

**Methods:**

TalaDME is a French, multicentric, two-arms, randomized, sham laser-controlled, double-masked trial evaluating the effect of photocoagulation of TelCaps combined to IVI in patients with ME associated with TelCaps. Patients with vision loss related to center involved ME secondary to RVO or DR and presenting TelCaps are eligible. Two hundred and seventy eyes of 270 patients are randomized in a 1:1 ratio to standard care, i.e., IVI of anti-VEGF solely (control group) or combined with IGTL therapy (experimental group). Stratification is done on the cause of ME (i.e., RVO versus diabetes). Anti-VEGF IVI are administered to both groups monthly for 3 months (loading dose) and then with a pro re nata regimen with a monthly follow-up for 12 months. The primary endpoint will be the number of IVI and the change in visual acuity from baseline to 12 months. Secondary endpoints will be the changes in central macular thickness, impact on quality of life, cost of treatment, and incremental cost-utility ratio in each groups.

**Key safety:**

Rare but severe AE linked to the use of IVI and laser, and previously described, are expected. In the sham group, rescue laser photocoagulation may be administered by the unmasked investigator if deemed necessary at month 3.

**Discussion:**

The best management of ME associated with TelCaps is debated, and there have been no randomized study designed to answer this question. Given the fact that TelCaps may affect 30 to 60% of patients with chronic ME due to DR or RVO, a large number of patients could benefit from a specific management of TelCaps. TalaDME aims to establish the clinical and medico-economic benefits of additional targeted laser. The results of TalaDME may raise new recommendations for managing ME and impact healthcare costs.

**Trial registration:**

EudraCT: 2018-A00800-55/ NCT03751501. Registration date: Nov. 23, 2018.

**Supplementary Information:**

The online version contains supplementary material available at 10.1186/s13063-024-07994-1.

## Introduction

### Background and rationale {6a}

The prevalence of diabetes in adults worldwide is estimated to reach 5.4% by 2025 [1] corresponding to a 300 million increase in the number of adults with diabetes worldwide. Among them, the prevalence of diabetic retinopathy (DR) is estimated to 35%. Diabetic macular edema (DME) represents the main cause of visual loss related to DR [2]. The prevalence of DME has been estimated at 7 to 10% among patients with DR [1–3]. Therefore, considering that there are 3.5 millions of diabetic patients in France, the prevalence of DME is estimated around 200,000 [4].

DME results from hyperpermeability of retinal vessels leading to an extravasation of plasma into the macula, the center of the retina. Until 2007, the only treatment used for DME was laser photocoagulation of the retina—RPE/photoreceptor complex—and/or of microaneurysms. The mechanism of visual improvement following laser remains unclear and the procedure is not yet standardized. Conventional laser halves the number of patients with VA loss after 3 years but provides limited visual improvement [5]. In the past decade, the development and the use of intravitreal injections (IVI) of anti-vascular endothelial growth factor inhibitors (anti-VEGF) or steroids allowed—for the first time—to achieve substantial VA gains in patients with DME. Intraocular steroids have however ocular adverse effects, including cataract and glaucoma, that limit their use in clinical practice [6]. Several large clinical trials have demonstrated the value of ranibizumab and aflibercept compared with laser in the treatment of DME [7, 8]. However, an iterative IVI scheme implies counterparts: a monthly visit schedule, the risk of endophthalmitis (0.02 to 0.1%), and the cost of treatment (drug, medical care and transport that represent, on average, several thousand euros per year and per patient). A median number of 15 anti-VEGF injections over 4 years is needed to achieve remission of DME [9–11] in 50% of cases. These studies have built up a consensus about the use of IVI as the first line therapy. The targeted photocoagulation of vascular lesions is not mentioned in the European consensus conferences for the treatment of DME [12, 13] and recommended only occasionally as a second-line treatment in the Preferred Practice Patterns from the American Academy of Ophthalmology (Retina Summary Benchmark 2021, AAO PPP Retina/Vitreous Panel, Hopkins Center for Quality Eye Care).

### Recent findings about the role of focal vascular abnormalities in macular edema

It has recently been shown that, during the course of RVO or DME, some microaneurysms may reach a size of several hundred microns [14]. A single macroaneurysm may cause such a severe breakdown of the blood-retinal barrier that a related severe macular edema may occur consequently. These were termed “capillary macroaneurysms” or more recently telangiectatic capillaries (TelCaps, 16) but may be also be part of the spectrum of the PEVAC (perifoveal exudative vascular anomalous complex) resembling lesions, occurring in association with vascular diseases, and described in diabetic retinopathy or retinal vein occlusion [15, 16]. TelCaps may be difficult to detect through routine imaging (i.e., SD-OCT, fluorescein angiography or OCT-A). Conversely, we have shown that indocyanine green angiography (ICG-A) combined with optical coherence tomography (OCT) improves the detection of these lesions [17]. Their role is therefore probably underestimated because ICGA is not routinely performed in ME patients and may be missed by ophthalmoscopy because of concomitant fundus changes (such as retinal hemorrhages, cotton-wool spots or hard exudates). Using ICGA, TelCaps have an estimated incidence of 30 to 66% in chronic maculopathy secondary to RVO or DR [18, 19].

**Fig. 1:**
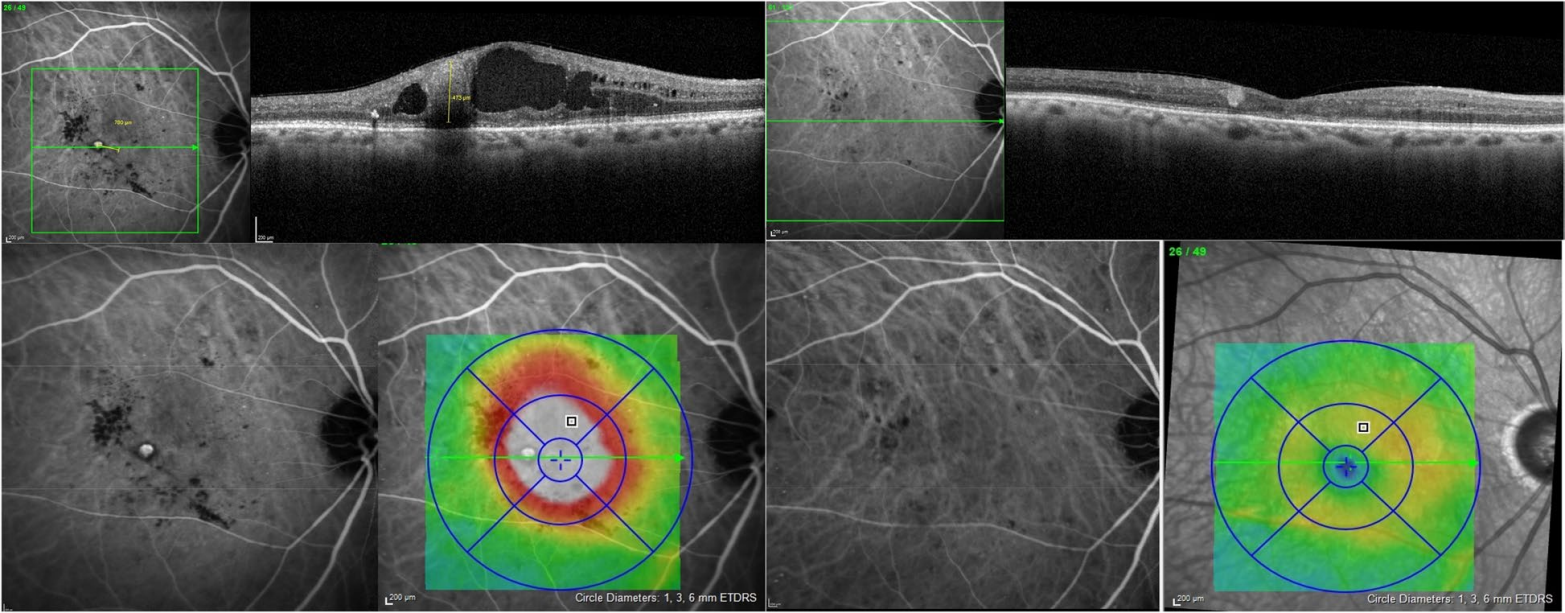
TelCaps before and after targeted laser photocoagulation. Left: A large TelCaps is visible on B-scan, with adjacent cystic edema, and characterized by a late ICG staining, located at the center of the macular thickening. Right: 2 months after targeted laser, the TelCaps is closed, leaving a hyper reflectivity on OCT B-scan. No ICG staining is visible, confirming the complete closure of the TelCaps, and associated with a complete resolution of macular edema

We subsequently developed a procedure termed indocyanine green-guided targeted laser photocoagulation (IGTL) which combines the detection of TelCaps by ICGA, laser photocoagulation by following, and immediate post-laser verification of the effectiveness of the photothrombosis by OCT. Our princeps publication [20] reported nine eyes suffering from longstanding macular edema (four eyes with DME and five eyes with RVO). Six months after photocoagulation alone, there was a significant reduction in the average macular thickness and an improvement in the best-corrected visual acuity (BCVA). Other studies subsequently confirmed our results [21–24]. It can therefore be hypothesized that, during the course of macular edema related to RD or RVO, the systematic detection of TelCaps by ICGA followed by their photocoagulation may be an effective treatment (Fig. 1).

We therefore believe that performing IGTL on TelCaps as an adjunctive treatment to intravitreal injections could lessen health costs and patient burden of ME management. The hypothesis is that part of the additional costs of laser would be offset by its long-lasting effects. A model-based economic evaluation of laser in DME found dominance or extended dominance in favor of laser associated with anti-VEGF compared with other options with and incremental cost-effectiveness ratio (ICER) of $12,410 per QALY [25].

### Choice of comparator {6b}

This study aims to compare laser combined to anti-VEGF vs anti-VEGF monotherapy. A systematic review and synthesis of the literature found that the management of ME results in a median number of 19 IVI of anti-VEGF over 5 years [26] in diabetics. This treatment has a heavy cost and requires a high frequency of follow-up visits. However, in real-life practice, patients treated with anti-VEGF are monitored less frequently and receive fewer injections than patients in clinical trials, resulting in lower VA gains than in pivotal studies [27]. In addition, the rate of lost to follow-up is high, reaching up to 30% at 5 years in diabetic patients with PDR treated with anti-VEGF [28]. Although dexamethasone implant usually allows to achieve a lower frequency of injections, the high incidence of cataract and the risk of glaucoma associated with the use of steroids, in diabetic patients—who are at higher susceptibility to develop glaucoma [29, 30]—limit their use.

The use of adjunctive conventional laser therapy over anti-VEGF therapy alone allows to moderately decrease the total number of anti-VEGF injections (median number of 13 injections vs 17 injections over five years using ranibizumab), with similar visual acuity gains [6, 7, 26].

### Evolving concepts of laser photocoagulation

Recent studies pointed out the relevance of phenotyping DME, differentiating focal from non-focal leakage on angiography [31], especially because the focal component may be less responsive to anti-VEGF therapy and consequently may benefit from adjunctive focal laser treatment [32].

A randomized study on the photocoagulation of microaneurysms (with most of them having a size inferior to 130 μm) did not found a clinical benefit [33]. On the opposite, as previously mentioned, we shown that targeted laser therapy alone—on lesions > 150 μm—was able to reduce DME at 1 year, with no additional injections, and was associated with vision gains, without the inconvenience, discomfort, and burden of recurrent anti-VEGF injections [20].

#### Objectives {7}

The objective is to demonstrate that IGTL, in association with standard of care by intravitreal anti-VEGF injections, allows significantly reducing the number of injections at 12 months of treatment versus absence of IGTL and is clinically non-inferior on visual acuity. The results of the study will be considered as positive for IGTL if both objectives are fulfilled.

#### Trial design {8}

The trial design is as follows: French, multicenter parallel group, 2-arms, randomized, sham laser-controlled, double-masked trial stratified on the cause of ME (RVO or DME).

## Methods: participants, interventions, and outcomes

### Study setting {9}

Patients will be recruited from tertiary care centers experienced with ME diagnosis and treatment. Patients will be screened during the selection visit that will consist of routine follow-up consultation with their ophthalmologist in the ophthalmology department of one of the participating centers: Quinze Vingts and Lariboisière (Paris), Saint-Denis (La Réunion), Bordeaux, Nantes, Pointe-à-Pitre, Dijon, Reims, Fort-De-France, Mantes la Jolie, and Bobigny.

### Eligibility criteria {10}

#### Inclusion criteria

**Fig. 2:**
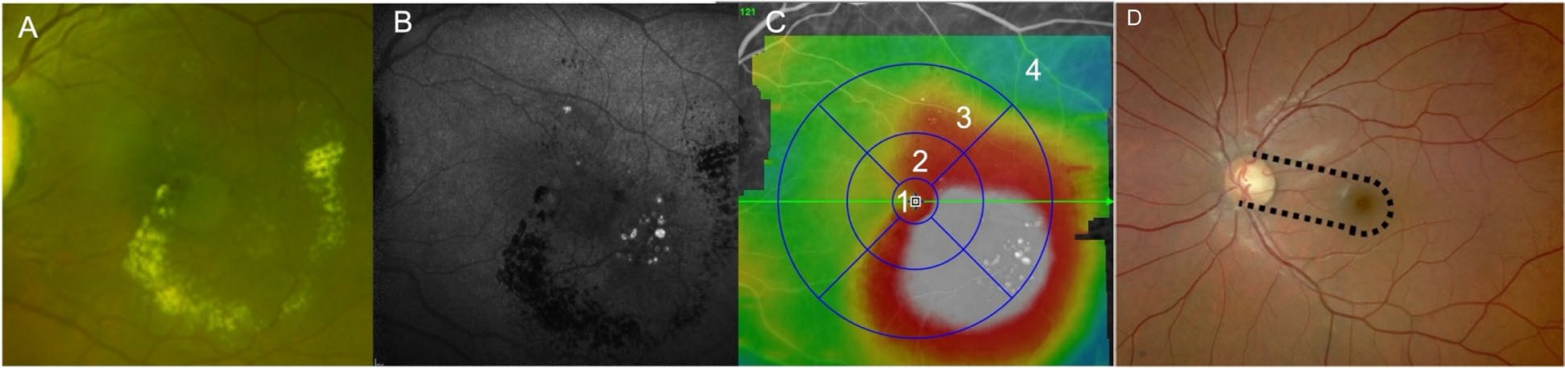
OCT map showing the 4 macular zones used to locate from the fovea and example of a TelCaps’ aggregate. **A** Color fundus photograph showing circinate exudates. **B** Late frame ICG (12 mn) showing multiple lesions located within a 1000-μm-diameter circle. Note the contrast between the background fluorescence and the TelCaps and the hypofluorescence of vessels. **C** ETDRS map showing the 4 macular zones used for TelCaps location. Zone 1: inside a 1-mm-diameter circle (< 500 μm of the center of the macula). Zone 2: inside a 3-mm-diameter circle (between 500 and 1500 μm of the center of the macula). Zone 3: inside a 6-mm-diameter circle (between 1500 and 3000 μm of the center of the macula). Zone 4: outside a 6-mm-diameter circle (beyond 3000 μm of the center of the macula. **D** Inter-papillomacular area (inside broken lines) represents the exclusion zone for photocoagulation

• Women and men ≥ 18 years.
• With BCVA lower or equal to ≤ 74 ETDRS letters (20/32 Snellen equivalent).
• With centro-foveal subfield thickness (CFST) of more than 300 μm in the central 1 mm of the ETDRS grid by SD-OCT corresponding to the normal value + 2 standard deviations: μm and/or presence of retro-foveal hard exudates (defined as the presence of exudates within the 1 mm diameter central ring of ETDRS grid).
• Due to ME secondary to DR or RVO.
• With at least one TelCaps with an individual diameter greater than 150 μm, located within a thickened retinal area, or aggregates of at least 3 late ICG-stained lesions, whatever their size, included within a circle of 1000 μm (= cluster), this circle being itself entirely located within a thickened retina (Fig. 2).
• With French Social Health Insurance.
• Who signed the written informed consent form.

In case of both eyes being eligible at the time of randomization, only one eye will be included (choosing the eye with the lowest VA). In case where both eyes have similar VA, the eye presenting with fewer TelCaps will be included. The fellow eye will be treated according to the site’s routine practice.

#### Non-inclusion criteria

Permanent:

- TelCaps mainly responsible for the ME located less than 500 μm from the center of the fovea (i.e., within 1 disc radius of the fovea), and without other eligible TelCaps, or TelCaps located in the inter-papillomacular area (Fig. 2D)
- Presence of age-related drusen or of age-related macular degeneration in any eye

Temporary (i.e., until condition changes):

- Significant opacity of the ocular media that could contribute to decreased visual acuity and/or impair laser realization. This temporary exclusion criteria will no longer be applicable once the patient has been operated on for cataract; a period of 6 months after cataract surgery will be required to be included in the protocol
- High-risk proliferative retinopathy requiring panretinal photocoagulation or associated with posterior tractional retinal detachment that may be worsened by the use of anti-VEGF therapy. This temporary exclusion criteria will no longer be applicable once the patient has been treated with PRP or operated on for PDR; a period of 6 months after PRP or surgery will be required to be included in the protocol
- Women who are pregnant, breast feeding, or of childbearing age without effective contraception
- Anti-VEGF injection in the past 4 weeks, cataract surgery within the last 3 months, myocardial infarction or stroke within the last 3 months, steroids intravitreal injection within the last 4 months. These temporary exclusion criteria will no longer be applicable once the patient has passed the above-mentioned delays

### Who will take informed consent? {26a}

The masked ophthalmologist will be responsible for obtaining the written informed consent from the patient. Patients are recruited through daily clinics, by hospital practitioners: either on a routine retinal check/screening for DR, or because they have chronic macular edema currently treated with IVI, or because they are addressed for a laser session in the context of a macular edema.

### Additional consent provisions {26b}

Additional consent provision is obtained by the masked investigator for collection and use of participant data and biological specimens. Participants consent to the collection of routine clinical data from their eye clinic after the study ends but can withdraw this consent at any time.

### Intervention description {11a}

The study visits will be performed on monthly intervals through 12 months. All examinations and assessments will be performed on both eyes. Maximum interval between screening visit and inclusion will be 30 days. If a patient has a ME with both diabetic retinopathy and RVO, the masked investigator will adjudicate if the ME is more imputable to DR or RVO.

Eligible participants will be randomized into one of the two study arms groups: “standard of care and sham laser” will consist of three monthly intravitreal anti-VEGF injections followed by additional injections in a PRN scheme with a monthly follow-up + sham laser (“control group”) or standard of care + IGTL (“experimental group”). Randomization will be stratified between RVO and DME.

To evaluate the interest of IGTL in conditions as close to real-life clinical practices, free choice of the anti-VEGF treatment used for intravitreal injection (ranibizumab or aflibercept) is given to each investigator. The anti-VEGF treatment administered to the patient should be tracked according to each center’s procedures.

*At the V0 visit*, the first laser treatment (IGLT or sham) will be administered within 5 days of first intravitreal injection.

*At the V3 visit* (i.e., after the initial three intravitreal injections), laser procedure will depend on imaging data.

- In the experimental group: if there are still TelCaps present on ICGA that require photocoagulation (among the ones selected at the V0 visit), then additional IGLT will be performed, whatever the CSFT. If no TelCaps are present on ICGA, then a sham laser will be performed to ensure masking.
- In the control group: *patients in the control group will undergo *another sham laser procedure*

*Rescue laser procedure {11b}: if patient in the sham group is non-responsive to anti-VEGF after 3 months (non-response being defined as a change in the CSFT of less than 20% from the baseline CSFT), then the unmasked laser investigator may decide to treat the TelCaps by IGTL. The patient will remain in the trial.

### Retreatment criteria by anti-VEGF injections

After the three initial injections of anti-VEGF, the patients will be followed up each month. At each consultation, a decision of retreatment by IVI on the basis of changes in VA and CFST will be made by the masked investigator. From V3 to V5, the injections will be given except if CSFT is < 315 μm or if the patient met the stability criteria. Stability corresponds to a change in CSFT ≤ 10% over the two previous visits or a change in VA ≤ 5 letters over the two previous visits. From visit V6, IVI will be suspended as long as stability is achieved. Treatment will be resumed if the CSFT increases by more than 10%, or if change in VA is > 5 letters, and retreatment will then be continued until a new stability is reached.

*At the V6 visit*, in case of non-response after six initial injections, either a switch to alternate anti-VEGF can be made, or the patient can be taken out from the trial in order to treat him/her with any treatment deemed necessary by the investigator, including steroids.

### Imaging procedures

*Color fundus imaging* will be carried out with the main aim of characterizing the presence and topography of dry exudates and determining the stage of diabetic retinopathy. Sites should use their routine device.

*Indocyanine green angiography* (ICGA) and *spectral domain optical coherence tomography* (SD-OCT) will be performed using the Spectralis© (Heidelberg Engineering, Heidelberg, Germany), which is the only marketed system for combined ICG and OCT angiography (Heidelberg Spectralis HRA + OCT, Heidelberg, Germany).

Typically, macroaneurysms appear between 30 s and 1 min after ICG injection, with gradual uptake over several minutes. The peak ICG uptake is usually observed after 5 min and the highest contrast over background (i.e., after the washout of dye) typically after 10 to 20 min. It is strongly recommended to also acquire images when the plasma fluorescence has faded, and hence the vessels appear as a shadow over the background retinal pigmentary epithelium fluorescence. Fluorescein angiography remains optional.

### *OCT procedure (*Fig. 3*)*

**Fig. 3:**
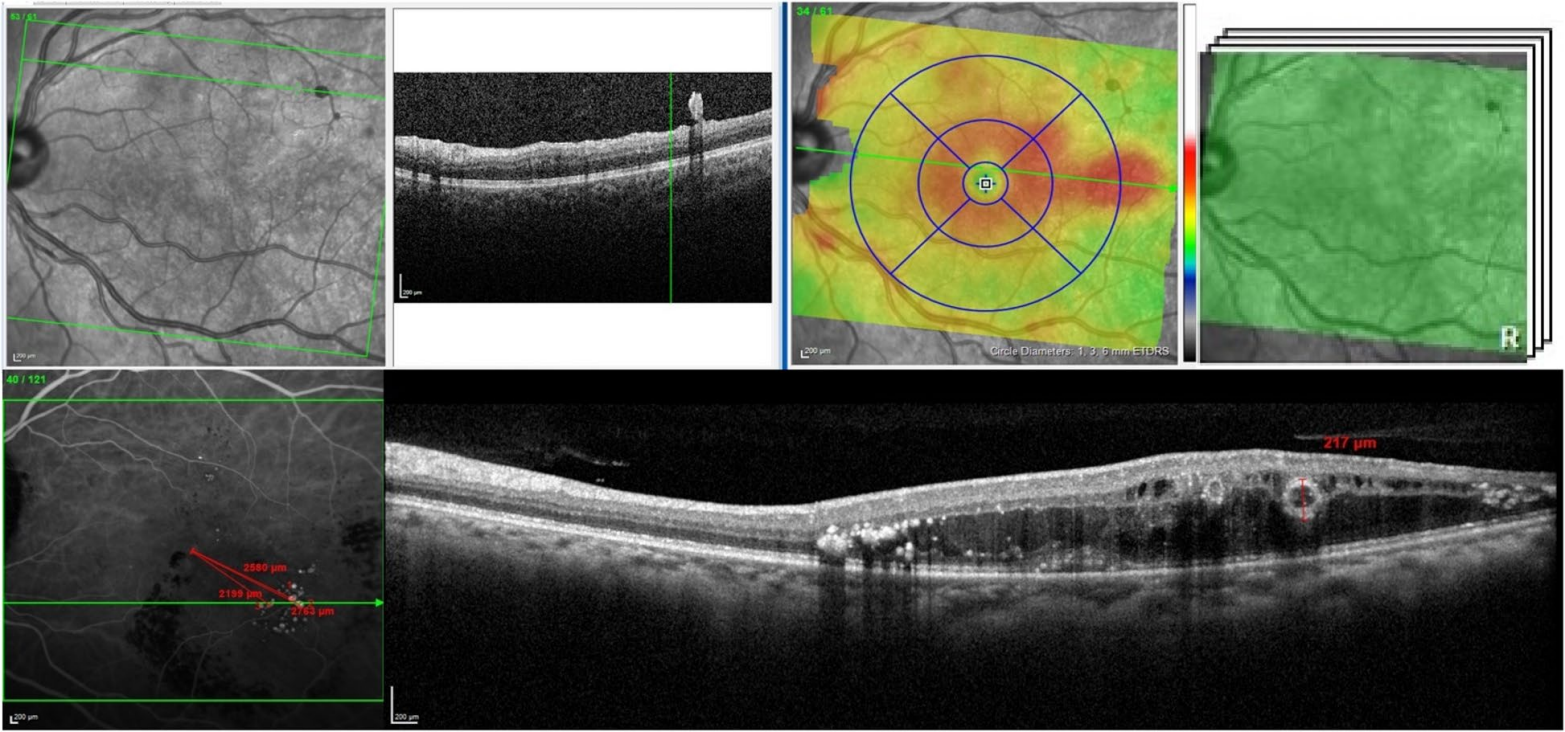
OCT acquisition protocol. Upper row: macular cube centered on the macula (30 × 25°, spacing minimal 60 and maximum 120 μm, HR, ART 1). Bottom row: Macular cube performed on the intermediate ICG frame ( 5 mn) with measurement of TelCaps size and distance to fovea

- Horizontal HD line 30° field
- Vertical HD line 30° field
- Raster scan centered on the macula (30 × 25°, spacing minimal 60 and maximum 120 μm, HR, ART 5)

### Measurements to be taken

• Number of single TelCaps or clusters; a cluster is defined by ICG uptaking lesions within a 1000μm circle count within a thickened retina (1 cluster counts for 1 TelCaps with a diameter of 1000 μm).
• Distance between the center of the lesions and the center of the fovea (Fig. 2). To evaluate the distance from TelCaps to fovea, the ETDRS grid centered on the fovea is used. Zone 1: inside a 1 mm-diameter circle (< 500 μm of the center of the macula); zone 2: inside a 3-mm-diameter circle (between 500 and 1500 μm of the center of the macula); zone 3: inside a 6-mm-diameter circle (between 1500 and 3000 μm of the center of the macula); zone 4: outside a 6-mm-diameter circle (beyond 3000 μm of the center of the macula.
• CFST (average thickness within the 1000 μm central ring of the ETDRS grid).
• Presence of retro-foveal exudates (defined as the presence of exudates within the 1 mm diameter central ring of ETDRS grid).
• Diabetic retinopathy severity.

### Targeted photocoagulation procedure

Laser photocoagulation will be performed using commercially available systems (532 nm or 577 nm, conventional monospot laser or multispot laser, equipped or not with an eytracker system). TelCaps will be identified by observation of the fundus and comparison to ICGA. The most common appearance of large TelCaps is that of a reddish spot surrounded by a whitish halo (the latter corresponding to the wall itself). Comparison with the ICG angiography images is useful to clearly identify the targets in uncertain cases (which is often the case if TelCaps are made of small aggregated lesions). The following parameters are suggested: size 50 μm; duration, 20 ms to 40 ms; power, 100 mW. The power will be increased until the operator visualizes a change in coloration of the TelCaps.

After the laser session, a new OCT scan may be immediately performed if deemed necessary, to check the efficacy of the laser procedure. This post-laser OCT check is recommended as it provides a quality check of the procedure. If performed, post-laser OCT imaging shall be done in automatic eye tracker mode with the pre-treatment as the reference image. The endpoint for photocoagulation is the presence of hyperreflectivity anywhere within the target TelCaps. If there is no evidence of energy transfer to the target, another photocoagulation session will immediately be performed.

At visit 3, ICG angiography will be performed. In the event of non-occlusion (i.e., persistence of staining of the TelCaps) and/or if there is persistence of retinal thickening, a new photocoagulation session will be proposed the same day and performed before intravitreal injection if this procedure is considered to be necessary.

#### Sham laser procedure

The patient is positioned for laser treatment in the same way; the physician will perform the same initial settings but without unlocking the laser. The aiming laser will be placed on the target or targets to be treated, and the laser will be activated approximately ten times.

### *Anti-VEGF therap*y

Ranibizumab or aflibercept may be used at the discretion of the investigator.

#### Prohibited treatments (medicinal, non-medicinal, surgical), including emergency treatment {11d}

The use of corticoid injections will not be allowed during the study in the studied eye.

#### Strategies to improve adherence to intervention protocols {11c}

Iterative treatments using intravitreal injections are known to be sometimes difficult to tolerate. Practitioners and CRAs will ensure that patients are systematically questioned about their ability to tolerate treatment and will encourage them to continue regular monitoring.

## Outcomes {12}

- ➢ Primary end points
  - Intensity of treatment estimated by number of anti-VEGF injections during the next 12 visits of treatment (over a maximum period of 14 months in case of delayed visit) that will be summarized by mean value in each group
  - Change in VA (letters) from baseline to the last visit, as measured by the Early Treatment Diabetic Retinopathy Study (ETDRS) that will be summarized by mean value in each group

Identification of two primary endpoints is justified by the fact that the study will be positive for the tested treatment if and only if BOTH tested hypotheses are demonstrated (reduction in number of anti-VEGF injections at 12 months are considered AND the non-inferiority on change in BCVA (Letters) from baseline to 12 months).

As recommended by the Committee for Proprietary Medicinal Products (CPMP) Guidelines, decision rule will be based on the upper bound of the 95% two-sided confidence interval (i.e., 97.5% one-sided) of the difference between the two groups for the changes in best-corrected visual acuity (DA SHAM – DA IGTL) will be less than the non-inferiority margin equal to five letters.

- ➢ Secondary end points
  - Change in retinal structure measured by change in central macular thickness between baseline and V12 and summarized by mean in each group
  - Cost of treatment over 12 months and incremental cost-utility ratio and summarized by mean in each group
  - Impact on quality of life: NEI VFQ-25 and EQ5D5L scores evolution between baseline and V12 and summarized by mean in each group
  - Safety of treatment estimated by the total number of adverse events (AE) and of serious adverse events (SAE) between baseline and end of study summarized and summarized by mean in each group and by proportion of patient with at least one AE or one SAE in each group."

### Participant timeline {13}

**Fig. 4:**
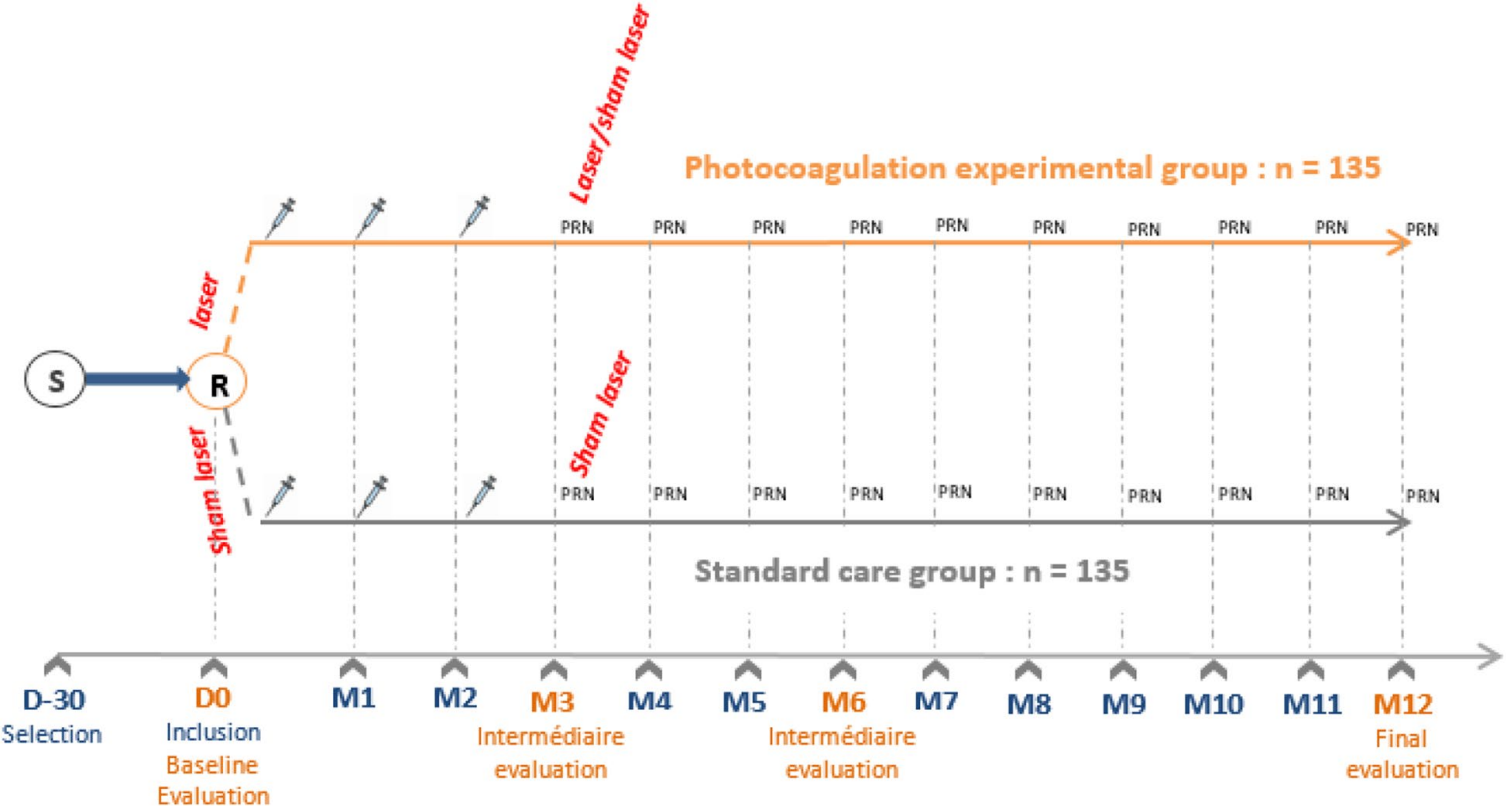
Scheme of the treatment arms

After inclusion, the visits will be performed on monthly intervals through 12 months:

- Standard follow-up should be performed within a window of ± 5 days of the scheduled visit date
- Evaluations visits (V3 V6 V12) should be performed within a window of maximum 30 days of the scheduled visit date. The schedule of procedures is shown in Table 1 and Fig. 4

**Table 1:** Schedule of visits and activities

Visits (± window)	V0	V1^b^	V2^b^	V3	V4	V5	V6	V7	V8	V9	V10	V11	V12
	Day1	1 month ± 5 days	2 months ± 5 days	3 months ± 15 days	4 months ± 5 days	5 months ± 5 days	6 months ± 30 days	7 months ± 5 days	8 months ± 5 days	9 months ± 5 days	10 months ± 5 days	11 months ± 5 days	12 months ± 30 days
Visit duration	2 h	1 h	1 h	3 h	1 h	1 h	3 h	1 h	1 h	1 h	1 h	1 h	3 h
Complete ophthalmologic examination (slit lamp examination, IOP measurement, and ETDRS BCVA)	✓	✓	✓	✓	✓	✓	✓	✓	✓	✓	✓	✓	✓
Blood pressure measurement	✓	✓	✓	✓	✓	✓	✓	✓	✓	✓	✓	✓	✓
Blood tests	✓^a^			✓^a^			✓^a^						✓^a^
Fundus photography	✓			✓	✓	✓	✓	✓	✓	✓	✓	✓	✓
OCT	✓	✓	✓	✓	✓	✓	✓	✓	✓	✓	✓	✓	✓
Indocyanine green angiography (**± **fluorescein angiography)	✓	✓	✓	✓			✓						✓
Quality of life questionnaire	✓^a^			✓^a^			✓^a^						✓^a^
Laser/sham laser	✓			✓									
Intravitreal anti-VEGF injection	✓	✓	✓	PRN	PRN	PRN	PRN	PRN	PRN	PRN	PRN	PRN	PRN

✓ Routinely performed

✓^a^Procedures specially performed for the need of the study

^b^During the induction phase (3 anti-VEGF loading doses), examinations on visits 1 and 2 are optional, except for anti-VEGF injection that remains mandatory

### Sample size {14}

Considering a standard deviation of the changes in visual acuity of 12 letters (i.e., conservative estimate issued from [34]), we would need 135 patients per group for a study power of 90% to demonstrate non-inferiority using a 97.5% one-sided confidence interval approach and considering a non-inferiority margin of changes in visual acuity equal to five letters. This margin was based on its pertinence regarding consequences for patients based on judgment of clinical experts.

In addition, using Cohen’s effect size definition and the methods proposed by Noether GE for sample size calculation for non-parametric tests, it can be calculated that this sample size will allow a 90% power to detect by a Mann–Whitney test a difference in the number of injections corresponding to a medium effect size at a 5% two-sided significance level.

In these calculations, it has been considered that up to 10% of patient of the laser group may be ineligible to laser realization due to technical issues (presence of media opacities or excessive uncontrolled eye movements or poor visibility of the macroaneurysms). All sample size calculations have been made using the NQUERY software (from Statsols USA).

### Recruitment {15}

Patients are recruited during routine ophthalmic consultations with their ophthalmologist in one of the eleven investigation centers. The centers participating in this study are experts for the management of macular edema and have experience in clinical research. Each center will have to recruit to 5 to 40 patients over a 54-month recruitment period.

## Assignment of interventions: allocation

### Sequence generation {16a}

The randomization is performed using a centralized on-line randomization system (CleanWeb). Randomization is stratified by center and by etiology (DME or RVO) without any restriction by stratum and used unequal blocked size.

### Concealment mechanism {16b} and implementation {16c}

The randomization list is inserted into the CleanWeb-based software and then forwarded to the sponsor’s quality assurance team for validation. The randomization is performed on the day of written consent obtainment by Web (CleanWeb) software, which assigns the patient a randomization number.

## Assignment of interventions: masking

### Who will be masked {17a}

The study is conducted in a double-masked fashion. The knowledge of the treatment group of the relevant patient will be kept to the absolute minimum of persons at the site and at the sponsor. The ophthalmologist practicing the lasers will be different from the one practicing anti-VEGF injections. This implies that each center designates two ophthalmologists for the duration of the study. The ophthalmologist performing anti-VEGF injections is blinded to the allocated group; hence, he/she will not perform laser.

### Procedure for unblinding if needed {17b}

In the case of medical emergency, unblinding of study treatment group for a participant may be necessary in the unlikely event that unblinding will guide further medical management to provide optimal treatment to the participant.

If unblinding is urgent and required to guide the immediate medical management of the participant, the investigator must call the clinical trial unit that is in charge of the management of the study and keep the randomization list. After checking the patient ID number in the data base and verifying that the requirement is justified by medical reasons, the information about the patient’s group will be provided to the investigator by a dedicated mail to ensure traceability of the transmission.

## Data collection and management

### Plans for assessment and collection of outcomes {18a}

All users of the study were trained and received a Certificate of Completion for Good Clinical practice. ETDRS BCVA will be determined after full refraction by masked, certified examiners using a certified room and certified equipment. The NEI VFQ-25 visual function questionnaire is a well-established, validated, patient-reported outcome measure (PROM) that has been used extensively in many trials. It is possible that loss of vision from ME has a particular impact on mental health. We therefore include the EQ5D5L questionnaire.

### Plans to promote participant retention and complete follow-up {18b}

Visits, investigator meetings, and regular newsletters will all aim to impress on sites the importance of retention.

As patients in the experimental group, compared with the control group, undergo a laser treatment that is part of the routine care, we expect to obtain a satisfactory retention rate.

Study activity feedback will be periodically collected from recruited participants to identify any arising or recurring issues with the consent and recruitment, screening, laser procedure, or follow-up activities. Centers are requested to perform the examination of the trial within a dedicated consultation to minimize the time spent by patients and facilitate the follow-up. Participants who choose to discontinue will be asked to complete an early withdrawal visit (which will include the same datapoints as for the study month 12 exit visit), and the reason for withdrawal will be documented.

### Data management {19}

Source documents for clinical data are based on paper or electronic patient files depending on the organization of each center and specific questionnaires are collected on paper. Data entry for data made non-identifying are carried out on electronic media via a web browser. The study is conducted using the CleanWeb® electronic data capture system. The e-CRF is protected by a system of secure access (login and password). Data-entry are made directly via internet following different profiles and saved in an Oracle database. Data transmission to the web-server are performed by means of an Internet connection, and no specific software has to be installed at the study sites.

Anonymity of the subjects is guaranteed by using a patient ID number on all documents necessary for research. A dedicated data manager is employed at F-CRIN platform (AP-HP, Paris, France) to guide the entire data collection and to provide technical assistance. Periodically, this data manager analyzes the data base to identify centers for which the delay in data entry or the rate of missing values for some variables are unacceptable or absence of consistency between variables. In addition, clinical research assistants dedicated by the sponsor will monitor data accuracy in the different centers.

### Confidentiality {27}

During or after research involving human subjects, the data collected on the research subjects and sent to the sponsor by the investigators (or any other specialized parties) are made pseudonymous.

Under no circumstances shall the names and addresses of the subjects involved be displayed on the trial documents.

Only the surname and first name initials are recorded, accompanied by an encoded number specific to the study indicating the inclusion order of the subjects.

The sponsor will ensure that each research subject has given written permission for access to personal information about him or her which is strictly necessary for the quality control of the study. Data processing follows the European General Data Protection Regulation (GDPR) and the additional specific French rules (CNIL, the French Data Protection Authority).

### Adverse event reporting and harms {22}

All AEs (related or unrelated to the study), whether reported by the participant, discovered during questioning, directly observed, or detected by physical examination, laboratory test or other means, will be recorded in the participant’s medical record and on the appropriate study-specific case report forms (CRF), stating the duration and intensity of the event, action taken by the investigator, and outcome of the event. CTCAE will be used to collected and/or analyzed AEs.

As diabetic patients have many comorbidities and a high risk of systemic complications, unrelated to their participation to the present study, we decided to exclude from the AEs the following situations:

- # Normal and natural evolution of the pathology:

Depending on the diabetes stage, patients may be hospitalized in a day-in unit or in the ward for a few days for the annual screening, for an acute glucose deterioration, or for the progression or for any diabetes complication (retinal detachment, coronary heart disease, acute kidney failure…).

Thus, normal or natural evolution of the study may lead to:

- Hospitalization scheduled for routine screening.
- Hospitalization for progression of a complication targeting the cardiovascular system, kidney function, painful neuropathy, foot ulcer.

- Hospitalization for glucose deterioration with or without a precipitating factor (such as infection)

#Special circumstances that do not need notification from investigators:

- Hospitalization for a pre-existing condition/disease.
- Hospitalization for medical or surgical treatment that has been scheduled before the study inclusion.
- Hospitalization for social or administrative reason.
- Hospitalization through the emergency room for diabetes or diabetes related-complications.

As regards laser therapy, retinal scars, increase of such scars with time, potentially involving foveal zone, and secondary choroidal neovascularization are the main expected complications. They will be studied using autofluorescence imaging from angiography frames at M3 M6 and M12 and using OCT B-scan study of external retina. In case of occurrence, they will be reported in the “comment item” located in the “imaging section” of the eCRF. Given prior experience of anti-VEGF therapy, the major adverse events expected are as follows: endophthalmitis, traumatic cataract related to IVI, retinal detachment, and potential increased risk of systemic adverse event such as acute myocardial infarction, cardiovascular disease, or kidney disease [35]. Those events will be collected and reported as SAE. The initial report, the SAE follow-up reports, and all other documents must be sent to the sponsor (by delegation the pharmacovigilance-CRO) by email to safety@fordrugconsulting.fr.

### Frequency and plans for auditing trial conduct {23}

To ensure the safety and respect of those individuals who have agreed to participate in the study, the sponsor will implement a quality assurance system to best monitor the running of the study in the investigation centers.

The *medico-economic evaluation* is of outmost importance given the economic burden of macular edema. Therefore, a health economics analysis will be performed as part of the study. Its objective will be to evaluate a strategy based on IGTL associated with anti-VEGF compared with anti-VEGF alone and to estimate the incremental cost per incremental quality adjusted life year (QALY). In non-inferiority studies, the cost-effectiveness analysis allows an appropriate representation of uncertainty, rather than hypothesis testing. We will represent the distribution of the joint density of mean cost and effect differences by using bootstrap replications. This removes the focus on hypothesis testing which leads to an overemphasis on type I errors and allows guidelines developers and policy makers to set their own thresholds for the probability that an intervention is acceptable [36]. This method of obtaining a confidence interval for cost-effectiveness from the cost-effectiveness plane and the acceptability curve does not give a confidence interval on the incremental cost-effectiveness ratio (ICER) statistic, as the ceiling ratio is defined only in positive quadrants of the cost-effectiveness plane. The statistical problems associated with negative ICERs (for example if the added targeted ICG laser therapy treatment is not superior but cheaper with reduced use of anti-VEGF) are avoided. Also, the results of the economic evaluation allow policy makers to set their own confidence level (not bound by the 5% alpha) and cost-effectiveness threshold.

The evaluation follows the recommendations from the French national health authority and the reporting will follow the CHEERS statement (https://www.has-sante.fr/jcms/r_1499251/fr/choix-methodologiques-pour-l-evaluation-economique-a-la-has/https://www.equator-network.org/reporting-guidelines/cheers). The perspective chosen is the healthcare system/payer and the patients, and the time horizon is 1 year. This choice is justified by the fact that studies in other countries have chosen the payers’ viewpoint; French patients with diabetic macular edema are eligible for 100% coverage of healthcare spending. Some patients however may elect to consult self-employed ophthalmologists during the study period and incur out of pocket costs for extra billing, which will not be captured. The economic evaluation is based on the entire population of patients included in the trial. Resources are collected prospectively at the patient level. The study is planned, undertaken, and analyzed according to the intention-to-treat principle. The unit of analysis is the patient. Because of the short duration of the follow-up, no discounting is required.

The costs of the laser will be estimated from microcosting for the procedure itself, collecting data on equipment and consumable use, staff time, and expected patient volume and usual rules for depreciation costs.

Resource utilization (we expect that patients will be managed as outpatients predominantly and that no hospital admissions will occur in relation to the protocol) will be collected at the patient level, partly via the study CRF (use of anti-VEGF and other treatment related to the edema) and partly via patient questioning during the follow-up visits. We will also ask patients about healthcare utilization for their eyes outside the study protocol and possible out of pocket payments.

Resource utilization related to adverse events will be systematically recorded.

All resources will be valued using the current list prices (drugs) and tariffs (consultations and tests). Hospital admissions will be valued using the current DGR costs.

The quality of life will be assessed in both groups at baseline, 3 months, 6 months, and at the end of the study. EQ 5D 5L scores will be valued using French tariffs [37]. The utility values will be attributed to the time period corresponding to mid-point between data collection. The primary outcome measure is cumulated QALYs. The utilities will be converted into QALYs for each arm using the area under the curve (AUC) method. This method assumes a linear relationship between values at different time points [38]. The gain of QALYs will be the difference between QALYs calculated in each arm. We will also compute the differential adjusted QALYs using regression models using the utility at baseline as an independent variable. The cost-effectiveness of IGTL combined to anti-VEGF vs anti-VEGF alone will be the incremental cost divided by incremental QALYs. The utility values will be attributed to the time period corresponding to mid-point between data collection. The difference in QALYs will be estimated as the difference in the area between the utility curves for the two treatment groups.

The economic endpoint is expressed as the point estimate of the incremental cost-effectiveness ratio (ICERs): where *Δ* costs (between groups)/Δ QALYs (between groups). In the case of non-inferiority studies, the innovation treatment is potentially decrementally cost-effective. In other words, the performance could be acceptably lower than reference strategy but result in a lower overall cost. If such a result is reproduced here, we plan subgroup analyses to identify which patients would be affected by a decrease in performance. The result is compared with the accepted French threshold values [39].

Baseline results will be presented as mean ± SD, median interquartile ranges (IQR), or as frequencies with percentages. Resource use data will be presented as means with standard error of the mean despite non-normal distribution because they better represent per patient data than median values and compared using nonparametric testing. Costs and QALYs will be presented as means with 2.5 to 97.5% bootstrapped intervals. Between-group comparisons of costs will be performed using the bootstrap *t*-test. Between-group comparisons of QALYs will be performed using nonparametric testing. A joint comparison of costs and QALYs will be performed by nonparametric bootstrapping with 1000 resamples. The uncertainty surrounding the ICER will be presented on the cost-effectiveness plane and acceptability curves.

All costs will be reported in € at the end of the study.

## Statistical methods

### Statistical methods for primary and secondary outcomes {20a, 20b}

The primary analysis will be conducted by the lead trial statistician, following the intent-to-treat principle where all randomized participants are analyzed in their allocated group, whether they receive the treatment they were allocated to following randomization. Baseline characteristics will be summarized for the two treatment groups. Continuous data will be summarized using means and standard deviations for data that follow anormal distribution or medians and interquartile ranges. Binary data will be reported as frequencies and percentages.

### Null and alternative hypotheses

The primary aim of the trial is to reject simultaneously two null hypotheses regarding:

- ➢ Number of anti-VEGF injections *N* during 12 months of treatment

This is a superiority hypothesis. The null and alternative hypotheses are as follows:

- H0: *N* IGTL = *N* SHAM.
- Versus.
- H1: *N* IGTL ≠ *N* SHAM.

- ➢ Change in best-corrected visual acuity (BCVA) (letters) from baseline to 12 months

This is a non-inferiority hypothesis that the IGTL group is non-inferior to the SHAM laser group; the null hypothesis will be that the difference between the two groups for the changes in best-corrected visual acuity (DA) will be less than the non-inferiority margin equal to 5 letters.

$$\text{H}_0:\;{\text{DA}}_{\text{SHAM}}\;-\;{\text{DA}}_{\text{IGTL}}\;\geq\;5$$

$$\text{H}_1\;{\text{DA}}_{\text{SHAM}}\;-\;{\text{DA}}_{\text{IGTL}}<\;5$$

Identification of two primary endpoints is justified by the fact that the study will be positive for the tested treatment if and only if both tested hypotheses are demonstrated (reduction in number of anti-VEGF injections at 12 months are considered and the non-inferiority on change in BCVA (letters) from baseline to 12 months). Since the two hypotheses should be simultaneously rejected, there is no adjustment of the nominal alpha value.

### Efficacy analysis

#### Main efficacy criteria

- ➢ Number of anti-VEGF injections *N* during 12 months of treatment
  Because of the nature of this variable, and to allow to be conservative in case of patients with extreme values, we will analyzed this parameter by non-parametric Mann–Whitney test.
- ➢ Change in best-corrected visual acuity (BCVA) (letters) from baseline to 12 months

As recommended by the Committee for Proprietary Medicinal Products (CPMP) Guidelines, decision rule will be based on the upper bound of the 95% two-sided confidence interval (i.e., 97.5% one-sided) of the difference between the two groups for the changes in visual acuity (DA SHAM – DA IGTL) will be less than the non-inferiority margin equal to five letters.

#### Secondary efficacy criteria

The following secondary endpoints of this study:

- ➢ Change in central macular thickness between baseline and V12
- ➢ Cost of treatment over 12 months and incremental (decremental) cost-utility ratio
- ➢ Impact on quality of life: NEI VFQ-25 and EQ5D5L scores evolution between baseline and V12 will be analyzed using non-parametric Mann–Whitney test

#### Handling of missing data {20c}

We will impute missing values using multiple imputations technics based on a Markov chain Monte Carlo (MCMC) method using PROC MI from SAS v9.4 and then use the MIANALYZE procedure from SAS v9.4 to generate valid statistical inferences [40].

#### Interim analyses {21b}

Interim analysis has been planned once half of the patients (i.e., *n* = 135) have reached their V12 visit. Reports concerning participants’ safety and key outcomes will be reviewed yearly by the steering committee.

### Data monitoring committee {21a}

The sponsor assigns clinical research associates (CRA) whose primary role is to carry out regular follow-up visits at the study locations, after the initial visits. It is responsible for the proper execution of the study, for collecting and documenting, and for recording and reporting the data generated in writing, in accordance with the Standard Operating Procedures applied within the CRID and in accordance with Good Clinical Practices as well as with the legislative and regulatory provisions in force.

#### Software

All analyses will be done by statisticians using SAS Version 9.4 (from SAS INSTITUTE). They will be blinded to the group of randomization that will be identified as group A or group B.

### Protocol amendments {25}

Any substantial modification to the protocol by the coordinating investigator must be sent to the sponsor for approval. After approval is given, the sponsor must obtain, prior to starting the study, approval from the CPP and authorization from the ANSM within the scope of their respective authorities.

The information sheet and the consent form can be revised if necessary, in particular if there is substantial modification to the study or if adverse reactions occur.

### Ancillary and post-trial care {30}

Many ancillary studies might be planned, using data from the present trial, especially on the natural history of TelCaps, the modulation of TelCaps by anti-VEGF IVI, semantic discussion around an overlap between PEVAC and TelCaps multimodal imaging of TelCaps, detection of TelCaps using OCT-mapping, and detection of TelCaps by AI. In case where further ancillary studies would be conducted, no provision will be allocated. When signing the consent form, patients agree to get their data retrospectively reused.

## Discussion

To the best of our knowledge, this is the first randomized, large-scale study to specifically address to the photocoagulation of large microvascular abnormalities in chronic vascular ME. In current recommendations for DME or RVO management from international ophthalmology societies, there is no mention of targeted laser while grid laser is being considered as a second-line therapeutic option [12]. A trial of targeted photocoagulation of microaneurysms smaller than 150 μm showed only modest clinical benefit [33]. However, it is interesting to note that only microvascular lesions identified by FA were included; TelCaps, which may be overlooked by FA, may hence have been missed. This justifies to reevaluate the effect of targeted laser in this indication, as several reports in the meantime have suggested that patients may benefit from targeted photocoagulation of TelCaps [20, 21, 23, 24, 41]. With TalaDME study, we expect to define a new standard-of-care for the subgroup of patients with vision loss related to TelCaps and hence to help allowing a better standardization of focal laser therapy. This study will imply optimized diagnosis and laser procedures that have not been evaluated in a trial yet. If these preliminary results are confirmed on a large group of patients, they may contribute to re-assess the role of photocoagulation in the management of chronic ME.

Defining the appropriate subgroup of candidates for targeted photocoagulation is a key issue. TelCaps do not have a consensual definition among the ophthalmologic community yet. TelCaps may show a rather large phenotypic spectrum, either presenting as isolated large bulges or aggregates of smaller lesions. There may be a continuum rather than a dichotomy between microaneurysms and TelCaps [42]. Besides, there might be an overlap between TelCaps and PEVAC (perifoveal exudative vascular anomalous complex) resembling lesions, occurring in association with vascular diseases, and described in diabetic retinopathy or retinal vein occlusion [15, 16]. OCTA and FA are of little interest for the positive diagnosis of TelCaps and are, hence, not considered for their positive diagnosis in TalaDME. Size of individual lesions based on OCT is a simple criterium, since TelCaps can be wider than the thickness of the normal retina, while microaneurysms are typically smaller than 100 μm. However, considering the size of individual lesions as a unique criteria may be too restrictive since we observed that very small, clustered lesions could be located in the center of ring exudates [18] (Fig. 2).

The most robust criteria for defining TelCaps appears to be late ICG staining. Based on the clinical experience of the PIs of the TalaDME study, TelCaps were defined as lesions > 150 μm showing prolonged, focal ICG staining located within a larger area of retinal thickening. Alternatively, TelCaps also includes aggregates of at least three lesions showing late ICG staining, whatever their size, included within a 1000-μm circle (= cluster), this circle being itself located within a thickened retina.

Thanks to the large database that will be obtained through the TalaDME study, several topics of research may be explored in ancillary studies. Because of the relatively recent identification of this entity, the diagnostic criteria are evolving. TalaDME will provide additional data to progress in this question. Retrospective analysis of the characteristics of responders and non-responders to laser in the present study will help to refine diagnostic criteria.

**Fig. 5:**
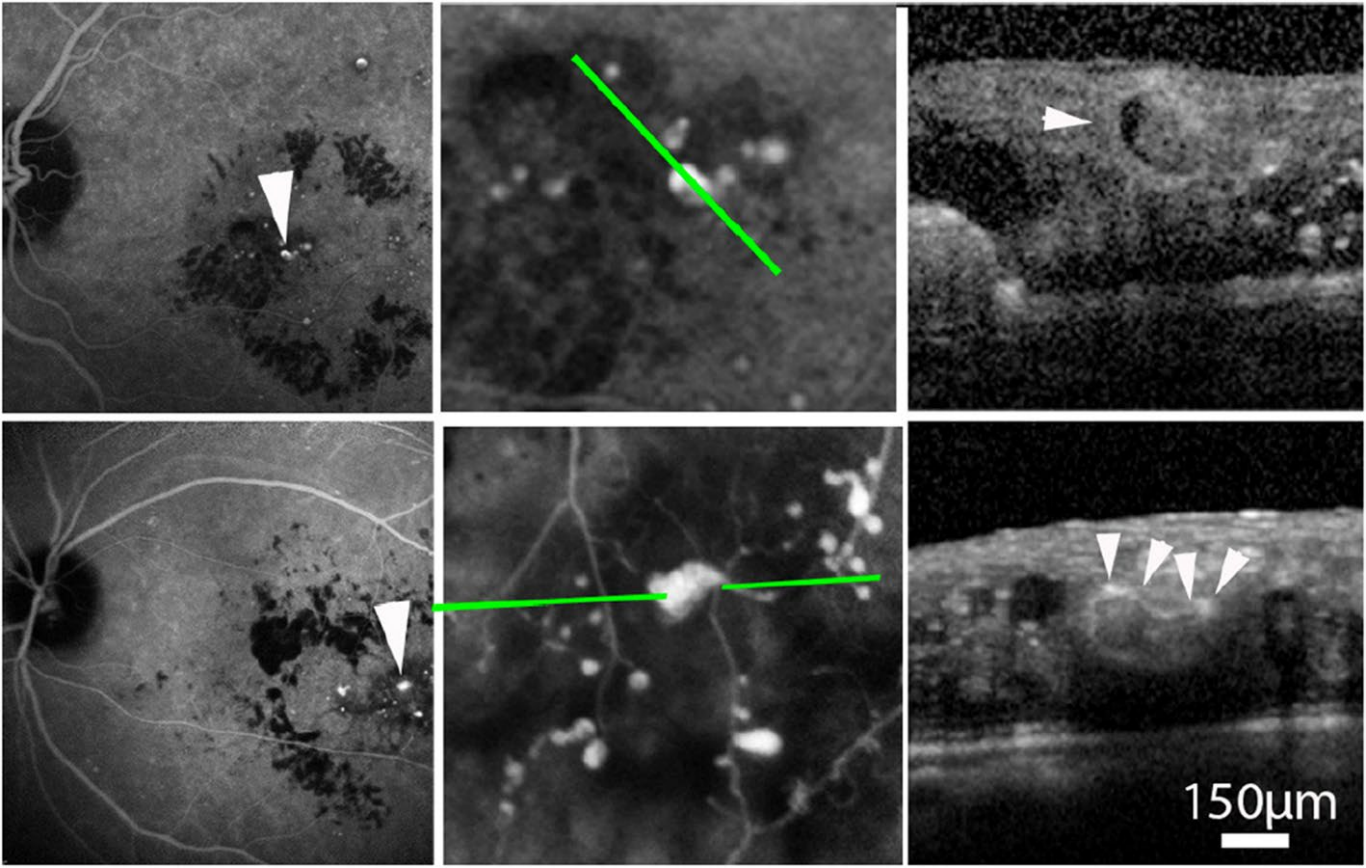
Illustration of different presentations of telangiectatic capillaries by optical coherence tomography. Top row, a 76-year-old man; bottom row, case 2, a 65-year-old man. Note the presence of intraluminal material (arrow), as a hypo-reflective croissant, narrowing the passage of blood flow (from Castro Farias D et al., Br J Ophthalmol 2019)

As ICG remains not available in many countries, defining ICG-independent criteria will be of importance for extending the use of targeted laser. Characterization of TelCaps using multimodal imaging will be helpful to better understand the pathophysiology of this entity and its various clinical presentations (Fig. 5). TelCaps are characterized by their functional characteristics, i.e., the strong, focal rupture of the blood retinal barrier (BRB) causing massive leakage and often lipid extravasation (hard exudates). As hard exudate often have a circinate pattern, it can be considered that vascular abnormalities at the center of circinate exudates are TelCaps. This provides some indications on the imaging characteristics of TelCaps. This shows that some TelCaps, taken individually, are relatively small and/or show a convoluted shape which cannot be defined by a single parameter such as size.

Laser modalities of TelCaps are evolving with technology. Recently, eye tracking has been implemented in some laser systems. This may improve the precision and safety of the procedure and decrease inter-operator variability.

We decided to associate a series of intravitreal injections after randomization in order to match to current treatment recommendations [12]. Nevertheless, this choice carries the risk of lessening the observed benefit of laser photocoagulation if laser is found efficient; however, not performing intravitreal injection would set a burden in the sham group which would have been left without therapy for several months. Future studies may evaluate the interest of skipping the three initial injections when performing IGTL.

### Trial status

Recruitment started on February 12, 2019, and closed on November 12, 2023. One hundred fifty-three patients have been included.

### Supplementary Information

**Additional file 1.**

**Additional file 2:** SPIRIT figure. Schedule of enrolment, interventions, and assessments.

## Data Availability

In accordance with GCPs: • It is incumbent upon the sponsor to obtain the permission of AQ all parties involved in the study to guarantee direct access to all locations where the study will be carried out, to the source data, and to the source documents and the reports, for the purposes of quality control and audit by the sponsor or inspection by the competent authority, • The investigators will make available to those in charge of monitoring, quality control, audit, or inspection of the study involving human subjects the documents and personal data strictly necessary for this control, in accordance with the legislative and regulatory provisions in force (Articles L.1121–3 and R.5121–13 of the Code de la Santé Publique).

## Abbreviations

AE: Adverse event
BRB: Blood retinal barrier
CFST: Centro-foveal subfield thickness
CM: Capillary macroaneurysms
CPMP: Committee for proprietary medicinal product
CRF: Case report form
CSFT: Central subfield thickness
DME: Diabetic macular edema
DR: Diabetic retinopathy
eCRF: Electronic case report form
EQ5D5L: EuroQol-5D questionnaire with 5-item vision bolt-on
ETDRS: Early treatment diabetic retinopathy study
FA: Fundus autofluorescence
F-CRIN: French clinical research infrastructure network
FRCRnet: French retinal clinical research network
ICG: Indocyanine green
ICGA: Indocyanine green angiography
IGTL: Indocyanine green-guided targeted laser photocoagulation
IVI: Intravitreal injection
ME: Macular edema
OCT: Optical coherence tomography
OCTA: Optical coherence tomography angiography
PDR: Proliferative diabetic retinopathy
PRN: Pro re nata
RPE: Retinal pigment epithelium
RVO: Retinal vein occlusion
SAE: Serious adverse event
SD-OCT: Spectral domain OCT
TelCaps: Telangiectatic capillaries
VA: Visual acuity
VEGF: Vascular endothelial growth factor
VFQ-25: 25-Item Visual Function Questionnaire
