# Supplementary figures and images for: Photocoagulation or sham laser in addition to conventional anti-VEGF therapy in macular edema associated with TelCaps due to diabetic macular edema or retinal vein occlusion (TalaDME): a study protocol for a multicentric, French, two-group, non-commercial, active-control, observer-masked, non-inferiority, randomized controlled clinical trial
Source: Trials. 2024 Apr 22;25:273. doi: 10.1186/s13063-024-07994-1 (PMC11034085; doi:10.1186/s13063-024-07994-1)

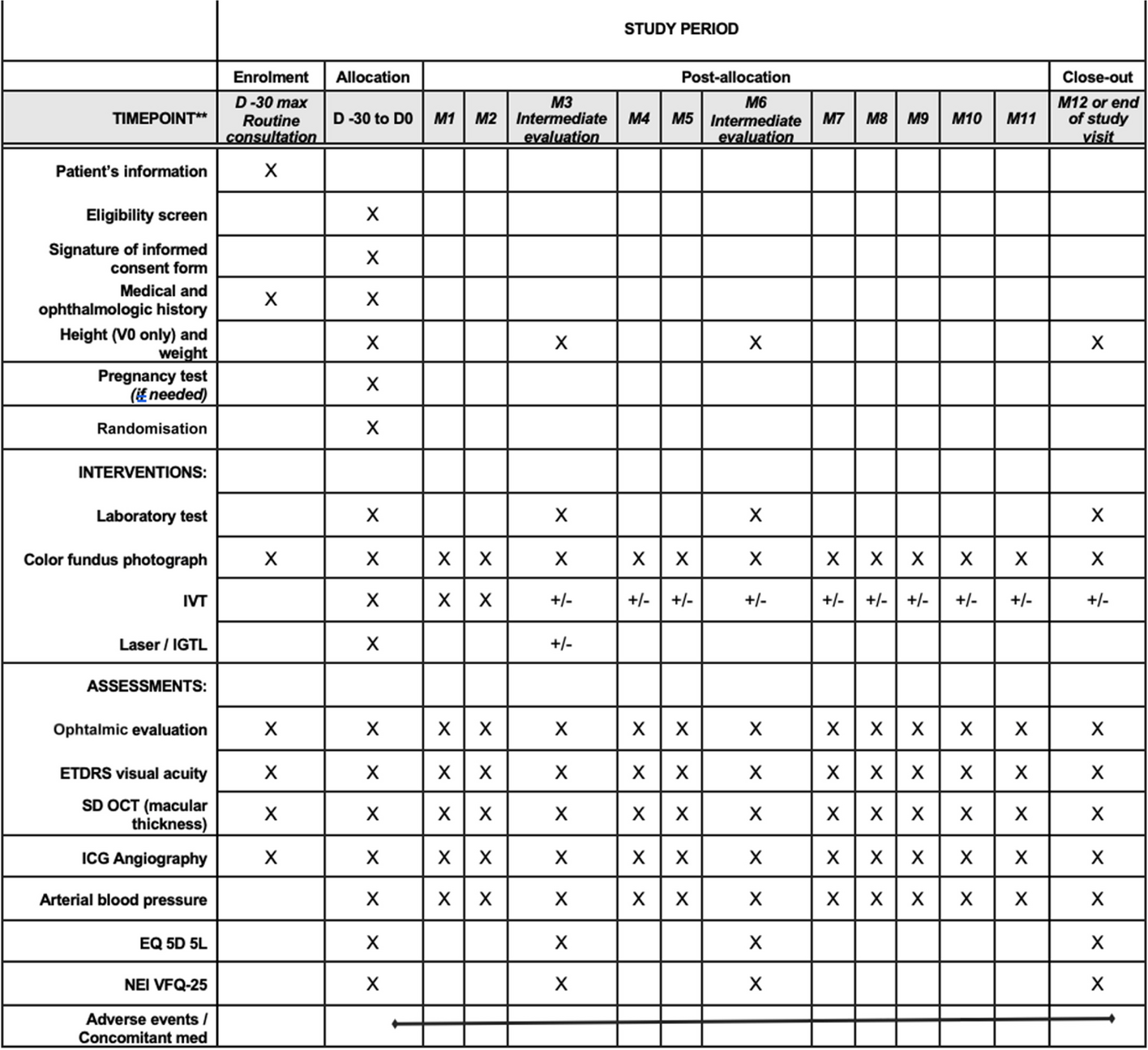

Supplement: Supplementary file 2 — Additional file 2: SPIRIT figure. Schedule of enrolment, interventions, and assessments. [file 13063_2024_7994_MOESM2_ESM.png]
